# Health-Related Quality of Life and Patients’ Empowerment in the Health Care of Primary Immune Deficiencies

**DOI:** 10.1007/s10875-017-0428-0

**Published:** 2017-08-17

**Authors:** Isabella Quinti, Federica Pulvirenti

**Affiliations:** 1 Department of Molecular Medicine, Sapienza University of Rome, Rome, Italy; 2 University Hospital Policlinico Umberto I, Viale Università, 37 00185 Rome, Italy

The health care of Primary Immune Deficiencies (PIDs) has always been challenging for clinicians due to the wide range of clinical phenotypes, difficulties in diagnosis, and complexity of treatments. Over the last decades, considerable progress has been made in awareness, prompt diagnosis, and therapy leading to a profound change in attitude and approach to primary antibody deficiencies (PADs). In particular, the advent of preparations of polyvalent immunoglobulin (IG) in the 1980s has dramatically improved morbidity and mortality. However, while IG replacement therapy has proven useful to control infections, other PADs-associated conditions related to chronic inflammation and cancer have not appeared to be improved by IG treatment. Disability in PADs is currently due to autoimmune complications, malignancies, recurrent gastrointestinal infections, and chronic lung involvement, with a strong impact on patients’ daily functioning [1]. The focus on the patients’ experience of illness requires a rigorous scientific approach to determine factors affecting the burden of disease to maximize patient’s well-being and to minimize the impact of disease.

The analysis of patients’ experience is surprisingly complex, and it is generally connected with the concepts of health-related quality of life (HRQoL), patients’ empowerment, and care satisfaction. Different instruments might be used in HRQoL studies such as generic questionnaires, disease-specific questionnaires, and treatment-specific questionnaires. Self-administered HRQoL questionnaires offer the most comprehensive representation of the patients’ perceived HRQoL. Such instruments quantify patients’ physical and psychological state, social interaction, well-being, and the individual’s perception of the impact of illness on their life [2]. Due to the extended life expectancy in PIDs, such assessments can provide pivotal details in pursuit of a functional balance between prolonging survival and preserving HRQoL [3]. Moreover, HRQoL outcome measures might help to assess disease progression over time and might also predict morbidity and mortality [4].

Since the first publication of HRQoL in PADs in 1993 [5], numerous studies have been published (review in 6). However, these studies involved small cohorts or were carried out mainly with the primary aim to compare different IG treatment schedules by the use of generic instruments such as short form (SF)-36 and SF-12 [7]. The generic questionnaires for HRQoL are constructed in such a way that they are intended—and validated—to be able to be used in all sorts of diseases, allowing the possibility to compare disease burden between different diseases and groups of patients.

The use of disease-specific tools is desirable to provide a more accurate picture of the burden of each disease. PID-specific questionnaires for HRQoL were validated many years ago [8]. Recently, new PADs-specific questionnaires have been validated [9, 10]. All surveys on HRQoL in PADs agreed that patients scored significantly lower when compared with age-matched normal controls and with patients affected by other chronic diseases, including cancer. Moreover, HRQoL declined over time, while the risk to develop psychological distress increased [4].

In the article in the current issue of the Journal of Clinical Immunology, Rider NL et al. [11] highlighted the impact of several clinical and individual factors influencing PADs HRQoL by analyzing the 12-item SF-12 questionnaire and the Immune Deficiency Foundation (IDF) questionnaire collected by IDF. This 75-question survey was developed by immunologists, experienced nurses, and patients, and it has been in use by the IDF for more than 30 years. Questionnaires were mailed to a large cohort of adult patients affected by Common Variable Immunodeficiencies (CVID) and had a good rate of response. Data analysis showed that a great number of CVID perceived to have a good disease control, even when their HRQoL scored lower on physical and mental domains in comparison to the US population. To be female, older, and having a late detection of disease or a permanent impairment in digestive and/or lung function were factors associated to decreased HRQoL and poor well-being. Moreover, the issue of fatigue in PADs is an emergent aspect raised by patients. One of the aims of Rider et al. was to assess the impact of the present standard of IG therapy on HRQoL in this vulnerable population. Those patients who reported the highest SF-12 scores were those who reported to have a well-controlled PAD, who were not bothered by treatment, and who received IG infusions at home. The authors showed that the route of IG replacement did not affect HRQoL. This is an interesting result in that the choice of IG schedule is crucial to determine adherence and satisfaction to replacement treatment. This decision is guided by several elements such as patients’ preference but also by clinical and social factors that could have an impact per se on well-being. However, the reasons for choosing the IG route, the definitions of PADs-associated complications, and the possible reasons for being “bothered” by IG infusions (adverse reactions versus inconvenience) were not addressed. Future longitudinal studies might help to better define the impact of IG treatment on HRQoL. In fact, patients treated at home could have a better HRQoL, because of a milder clinical condition, while the hospital setting could have been proposed to patients with a more serious disease and/or a disadvantaged social condition. Moreover, it might be necessary to analyze the correlation between the infusion interval and the occurrence of periods of fatigue or low energy between IG administrations. At the same time, the severity of organ impairment linked to perceived health should be established by objective medical records. With these limitations, the publication of such a large survey done by patients on their HRQoL is relevant and stimulating for immunologists involved in PIDs care who need to know the patients’ perspectives on health outcomes.

Even if originally developed to assess treatment effectiveness in clinical research, patient reported outcome measures are now increasingly used in clinical routine to improve the care for individuals and in health management and policy. To truly capture the patients’ perspective, patients’ involvement in the tool development is crucial to identify the items, discussing their relevance and verifying the comprehension of the wording. As attempted by the IDF study, this approach needs the patients’ contribution for the recognition of patients’ beliefs, needs, and preferences in the shared decision-making process to improve adherence to the treatment, to ameliorate disability, and to favor clinical encounter in everyday medical contexts.

We, doctors and nurses working with patients affected by PIDs, should recognize that the patients’ empowerment is a determining factor in care. The introduction and the regular use in our clinical practice of validated instruments developed by patients to assess their HRQoL is going in the right direction.
